# Apical Tomato‐Based Seasoning Powder: Physicochemical Characterization, Antioxidant Activity, Aromatic Compounds, and Sensory Analysis

**DOI:** 10.1111/1750-3841.71610

**Published:** 2026-11-11

**Authors:** Márcia Aparecida de Souza, Maria Gláucia Dourado Furquim, Viviane Patrícia Romani, Julio Colivet Briceno, Bruna Melo Miranda, Tatianne Ferreira de Oliveira, Abadia dos Reis Nascimento

**Affiliations:** ^1^ Federal University of Goiás Samambaia Campus Goiânia Goiás Brazil; ^2^ Federal Institute Goiano Iporá Campus Iporá Goiás Brazil; ^3^ Federal Institute of Paraná Palmas Campus Palmas Paraná Brazil

## Abstract

Many fruits at the plant apex (apical tomatoes) are discarded during table tomato production, contributing to losses reaching one‐third of global food production. This study aimed to develop a seasoning powder using apical tomatoes to reduce waste and add value, as seasonings can be effectively used to improve the palatability of foods. Five formulations were produced containing the following percentages of apical tomato: F1 (0%), F2 (15%), F3 (35%), F4 (55%), and F5 (70%). Samples were evaluated for physicochemical properties, phenolic compounds, antioxidant activity, aromatic profile, microstructure, microbiological safety, and sensory acceptance. F5 showed the highest phenolic content (31.06 mg gallic acid equivalent [GAE]/g), antioxidant activity (0.38 µM/g), more intense red and yellow coloration, and the greatest sensory acceptance. It also presented 40 aromatic compounds and the highest hygroscopicity. All formulations showed satisfactory results in microbiological analysis and met the standards of the legislation. Results indicate that apical tomatoes are promising for producing a natural, functional, and sustainable seasoning powder with commercialization potential.

## 1 Introduction

Table tomatoes (*Solanum lycopersicum* L.) are one of the most consumed vegetables in the world, standing out for both their economic value and their nutritional profile, especially for the presence of bioactive compounds such as carotenoids, phenolics, and antioxidant vitamins (Ali et al. 2021; Dorais et al. 2008). In the context of production intended for fresh consumption, a significant fraction of fruits located at the apex of the plant, popularly called apical tomatoes, are not harvested when the market value does not offset the operational costs of harvesting and logistics. These fruits, although physiologically fit for consumption, show variations in size, color, and uniformity that limit their acceptance by the final consumer, resulting in underutilization in the production chain. Studies indicate that these fruits maintain a relevant chemical composition, including high levels of lycopene, organic acids, and volatile compounds, which highlights their potential for alternative technological applications (Agarwal and Rao 2000; Toor and Savage 2005).

Seasonings and spices play a central role in the food industry because they simultaneously contribute to sensory improvement, oxidative stability, and, in some cases, to the functional value of products (Feng et al. 2018; Viuda‐Martos et al. 2011). The literature records several studies aimed at the development of seasoning mixtures based on vegetables, aromatic herbs, and spices, with emphasis on formulations rich in phenolic compounds and natural antioxidants, capable of replacing synthetic additives (Okonkwo et al. 2024; Shan et al. 2005; Embuscado 2015). However, despite the extensive exploitation of ingredients such as garlic, onions, peppers, Mediterranean herbs, and plant by‐products, there is a scientific gap regarding the development of seasonings made specifically from discarded table tomatoes, especially those from the plant apex. To date, there are no consolidated reports in the literature that address the systematic use of apical tomatoes as the main matrix for the formulation of seasonings with physicochemical, antioxidant, and aromatic characterization.

Reducing food waste and using underutilized fractions of agricultural production are priority challenges for sustainable food systems. It is estimated that approximately one‐third of the food produced globally is lost or wasted along the production chain, with significant economic, environmental, and social impacts (FAO 2019). In this context, the use of fruits that are discarded still in the primary stage of production represents a promising strategy for the circular economy, especially when associated with the development of higher value‐added ingredients. In the case of tomatoes, studies have shown the potential of residues and fruits outside the commercial standard to obtain products with high antioxidant content, such as powders, extracts, sauces, and functional ingredients (Sagar et al. 2018 Szabo et al. 2025). However, most of these investigations focus on industrial waste, so the direct use of fruits discarded in the field from the producer's perspective remains little explored.

In view of this scenario, the present study aimed to develop a seasoning using apical tomatoes that would be discarded in the production of table tomatoes and evaluate their physicochemical quality, antioxidant activity, profile of aromatic compounds of the final product, and sensory analysis. The differential of this study lies in the valorization of a material that is still little explored scientifically, integrating the reduction of losses at the base of the production chain with the development of a product aligned with the demands for natural, sustainable, and sensorially attractive ingredients. By directing the use of apical tomatoes to the development of a seasoning with technological and functional potential, this study contributes to the expansion of knowledge about the valorization of agricultural waste and offers scientific support for value‐adding strategies from the perspective of the producer and the food industry.

## 2 Materials and Methods

This research was carried out in the Vegetable Garden of the School of Agronomy (EA) of the Federal University of Goiás (UFG), using the surplus of tomatoes. Physicochemical and chromatographic analyses (gas chromatography–mass spectrometry [GC–MS]) were conducted at the Food Engineering Laboratory of EA/UFG, whereas microbiological analysis was performed at the Food Research Center (CPA/UFG), and scanning electron microscopy (SEM) was performed at the Regional Center for Technological Development and Innovation (CRTI/UFG).

### 2.1 Obtaining Raw Material

The tomatoes used in the research were grown in the Vegetable Garden of the School of Agronomy of UFG (16°35′46.6″ S 49°16′52.7″ W) from April to September 2023. Planting was carried out in an open field, with staking, using one plant per hole, at a spacing of 1.0 × 0.60 m, as recommended by Trani et al. (2015). During the vegetative period, the plants were irrigated regularly to ensure proper development. The fruits used were apical tomatoes, that is, those located at the apex of the plant, of the Zenzei variety. Harvest was carried out 174 days after planting in the field.

### 2.2 Seasoning Formulation

Seasoning development began with the production of tomato powder. This process consisted of the stages of receiving the tomato, washing and sanitizing, cutting, drying in an oven, crushing, mixing the ingredients, and packaging. After the initial stages, the fruits were cut into slices of uniform thickness and dried in an oven with air renewal and circulation (TE‐394/3, Tecnal) at a temperature of 57°C for a period of 46 h. After drying, the tomatoes were crushed in a semi‐industrial blender and subsequently sifted.

With the tomato powder obtained, the seasoning began to be formulated using different proportions of the ingredient as a base. Five formulations were developed containing different proportions of tomato powder: F1 (0%), F2 (15%), F3 (35%), F4 (55%), and F5 (70%). The other components of the formulation were maltodextrin, which varied in the different formulations [F1 (70%), F2 (55%), F3 (35%), F4 (15%), and F5 (0%)], salt (20%), paprika (3.5%), ascorbic acid (2%), garlic (1.5%), onion (1.5%), black pepper (0.5%), red chili pepper (*Capsicum baccatum*) (0.5%), and herbs (rosemary, basil, and oregano) (0.5%), in the same proportions for all formulas. Analyses were carried out to determine the physicochemical, aromatic, microbiological, and sensory acceptance of the product, as described below.

### 2.3 Density, Solubility, and Hygroscopicity

To determine the density, 10 g of sample was weighed on a precision scale and transferred to a 25 mL graduated cylinder, where the volume was measured. Density calculation was performed using the formula suggested by Rahman (2009), which defines density as the ratio between the mass and volume of a substance and is expressed in g/cm^3^.

To determine solubility, 1 g of each sample was diluted in 30 mL of distilled water and stirred for 30 min at room temperature. Then, the samples were centrifuged at 5000 rpm for 10 min, and the supernatant was separated. The residue was dried in an oven at 105°C until it reached constant weight. Weighing was carried out before and after the process (Huang et al. 2019). Solubility was obtained as the ratio between the weights of the dry sample and the natural sample multiplied by 100 and expressed as a percentage (%).

A quantity of 8 g of each sample was weighed and dried in an oven at 50°C until reaching constant weight to determine hygroscopicity. A saturated solution was prepared using 36 g of sodium chloride in 100 mL of distilled water and kept at rest for 24 h in the containers with the samples. Then, 2 g of sample was deposited in disposable plastic cups, which were placed in a desiccator with controlled relative humidity at 75%. The samples were periodically weighed at intervals of 12–156 h for monitoring. Each sample was weighed in triplicate to obtain the average (Aslan and Ertas 2021), according to the following equation, where *M_i_* is the mass of the sample after water absorption every 12 h (g), and *M*_0_ is the initial mass of the seasoning (g):

$$\mathrm{Hygroscopicity}\;\left(\%\right)=\frac{M_{i}-M_{0}}{M_{0}}\times 100$$

### 2.4 Colorimetry

Color analysis was performed on a Shimadzu UV‐1800 spectrophotometer to measure the amount of light absorbed by the apical tomato powder samples and the seasoning formulations. The variables analyzed by the CIELAB system are color lightness (*L*), ranging from 0 (black) to 100 (white), which measures color clarity or darkness; the chromaticity coordinate a*, which indicates color position between green (−*a*) and red (+*a*); and the chromaticity coordinate b*, which refers to color position between blue (−*b*) and yellow (+*b*) (Seibel et al. 2022). The total color difference (Δ*E*) was calculated as the following equation:

$$\Delta E=\sqrt{{\left(\Delta L\right)}^{2}+{\left(\Delta a^{*}\right)}^{2}+{\left(\Delta b^{*}\right)}^{2}}$$

### 2.5 Phenolic Compounds and Antioxidant Activity

Quantification of phenolic compounds was performed by spectrophotometry at 750 nm, using the Folin–Ciocalteu reagent (Waterhouse 2002). Phenolic compounds were determined in the aqueous extract, and their quantification was based on a standard curve of gallic acid. The results were expressed in milligrams of gallic acid equivalent (GAE) per 100 g of sample.

Antioxidant activity was determined by the DPPH (2,2‐diphenyl‐1‐picrylhydrazil) radical scavenging method. This method is based on the reduction of the DPPH radical, which, by fixing an H (removed from the antioxidant under study), leads to a decrease in absorbance at 517 nm. A calibration curve was constructed using the antioxidant agent Trolox (6‐Hydroxy‐2,5,7,8‐tetramethylchroma‐2‐carboxylic acid) as a reference. The results were expressed with respect to the curve, as antioxidant activity (µM Trolox/g sample) (Waterhouse 2002).

### 2.6 Gas Chromatography–Mass Spectrometry

GC–MS analysis was performed on a Shimadzu gas chromatograph model GCMS‐QP2020 NX, coupled to a mass spectrometer, with an AOC automatic injector and equipped with an SH‐Stabilwax‐MS column (30 m × 250 µm, 0.25 µm). To achieve the same sample volume in the headspace vials, 2.46 g of F0 (apical tomato powder), 3.51 g of F1, 2.08 g of F2, 2.11 g of F3, 2.23 g of F4, and 1.90 g of F5 were weighed. The initial temperature of the oven was 40°C for 5 min, then increased to 160°C at a rate of 5°C/min, then to 160°C, and finished at a rate of 10°C/min at 250°C, remaining for 15 min. The analysis time was 52 min. The injection volume was 1000 µL in 1:10 split mode, and the helium carrier gas was adjusted to a constant flow rate of 0.94 mL/min and a linear velocity of 35.0 cm/s. The temperature of the injector, interface, and ion source was maintained at 250°C. The methodology used was adapted from Alves et al. (2023). For presentation and evaluation of the results, the associations between the aromatic compounds and the aromas described in this study were checked against the Flavornet database (Acree and Arn 2004).

### 2.7 Scanning Electron Microscopy

The images for studying the structure and morphology of the samples were obtained with a Jeol JSM‐IT300LV SEM with an electron acceleration voltage of 15 keV in the backscattered electron detection (BED) mode at the Regional Center for Technological Development and Innovation—CRTi of the Federal University of Goiás.

### 2.8 Microbiological Analysis

Microbiological analysis was carried out at the Food Research Center—CPA of the Federal University of Goiás. The ISO 16649‐2:2001 method was used for the count of *Escherichia coli*, and the ISO 6579‐1:2017/Amd 1:2020 method was used for the count of *Salmonella* spp. Both methods were conducted according to Normative Instruction (NI) No. 161, of July 1, 2022, of the National Health Surveillance Agency (ANVISA). All samples were analyzed right after their production.

### 2.9 Sensory Analysis

Sensory analysis was carried out at the Laboratory of Sensory Analysis of the Food Engineering Department of UFG, with 100 assessors, according to the Adolfo Lutz Institute (2008). The study was reviewed and approved by the UFG University IRB under approval #6.901.897, and informed consent was obtained from each subject before they participated in the study.

The formulations were evaluated using the seasonings in instant noodles. Samples of 15 g of noodles at a temperature of around 35–40°C containing the developed seasonings were provided. In the preparation, 2.5 g of seasoning was used for every 100 g of noodles. The samples were coded and randomly distributed for evaluation. Water was provided to the assessors to cleanse the palate after tasting each sample. The samples were analyzed based on a 9‐point hedonic scale for evaluation of appearance, odor, texture, and taste, where 1 is “extremely disliked,” and 9 is “extremely liked.” Purchase intention was also evaluated on a 5‐point scale, with 1 corresponding to “probably would not buy” and 5 corresponding to “probably would buy.”

### 2.10 Statistical Analysis

Statistical analysis was performed using Statistic 7.0 software. Analysis of variance (ANOVA) of the data obtained was performed, and the Tukey test at a 5% probability level was used to compare the means.

## 3 Results and Discussion

### 3.1 Density and Solubility

The density of the formulations ranged from 0.47 to 0.63 g/cm^3^ (Table 1), and the samples with the highest tomato powder percentage had lower densities. A significant difference (*p* < 0.05) was observed only between F0 (100% tomato powder) and F1 (0% tomato powder), whereas the other samples did not show significant differences (*p* > 0.05). The increase in density may be related to the presence of other ingredients that contributed to making the seasoning heavier. The density of this seasoning is close to that of garlic, which is a condiment widely used in Brazilian cuisine. Poojitha and Athmaselvi (2023) found a density of 0.83 g/cm^3^ for garlic powder, within a similar range reported in the present study.

**TABLE 1:** Density and solubility of apical tomato powder and seasoning formulations (*n* = 3 ± SD).

Sample	Density (g/cm^3^)	Solubility (%)
F0	0.47 ± 0.01^c^	66.75 ± 0.68^f^
F1	0.63 ± 0.07^a^	94.32 ± 0.34^a^
F2	0.61 ± 0.01^ab^	87.58 ± 0.39^b^
F3	0.56 ± 0.02^ab^	79.52 ± 0.97^c^
F4	0.54 ± 0.01^bc^	76.65 ± 0.86^d^
F5	0.53 ± 0.01^bc^	72.30 ± 0.25^e^

*Note*: F0 = apical tomato powder; F1 = formulation containing 0% apical tomato; F2 = formulation containing 15% apical tomato; F3 = formulation containing 35% apical tomato; F4 = formulation containing 55% apical tomato; F5 = formulation containing 70% apical tomato. Different letters in the same column indicate significant differences (*p* < 0.05) between the samples by Tukey test.

Regarding solubility, all samples were considered statistically different (*p* < 0.05). Values ranged from 66.7% (F0—100% tomato powder) to 94.32% (F1—0% tomato powder). In terms of food, a higher solubility is generally desirable because it indicates that nutrients or bioactive compounds can easily dissolve in liquids. This is a desirable characteristic for seasonings as they play a crucial role in cooking, and their solubility can affect the taste, texture, and appearance of dishes (Yi, Jia and Li 2024).

### 3.2 Hygroscopicity

Understanding the hygroscopic behavior of food is essential to define the appropriate conditions for dehydration, packaging, and storage of the product (Labuza and Altunakar 2007). Hygroscopicity evaluation was performed over a period of 358 h (approximately 15 days) and revealed an increase over time for all formulations (Figure 1), indicating that the seasonings absorb moisture from the environment. Formulation F5 had the highest hygroscopicity at the end of the experiment (143.83%), whereas formulation F0 had the lowest value (97.36%). The other ingredients added to the seasoning, in addition to the apical tomato, contributed to an increase in the hygroscopicity of the samples. This increase in hygroscopicity is an important factor to consider when storing seasonings, as it refers to the ability of a material to absorb moisture from the environment, which can affect product texture, flavor, and especially durability, in addition to serving as an indicator of the type of packaging to be used.

**FIGURE 1:**
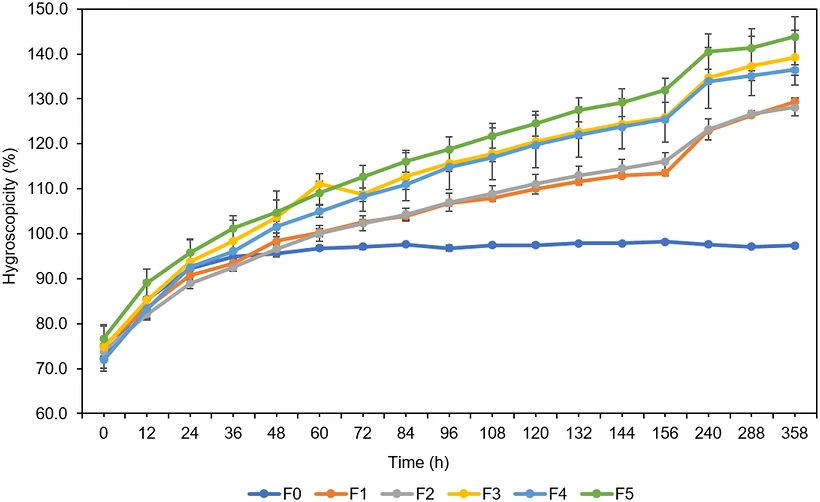
Hygroscopicity of apical tomato and seasoning formulations (*n* = 3 ± SD). F0 = apical tomato powder; F1 = formulation containing 0% apical tomato; F2 = formulation containing 15% apical tomato; F3 = formulation containing 35% apical tomato; F4 = formulation containing 55% apical tomato; F5 = formulation containing 70% apical tomato.

### 3.3 Total Phenolic Compounds and Antioxidant Activity

Total phenolic compounds are widely found in a variety of foods and are known for their antioxidant properties and potential health benefits. Studies suggest that phenolic compounds may help fight oxidative stress in the body, reducing the risk of chronic diseases, such as cardiovascular disease and cancer (Cory et al. 2018). Figure 2 illustrates the amount of total phenolic compounds in tomato powder and seasoning formulations. A significant difference (*p* > 0.05) in phenolic compounds was observed between F0 (100% tomato powder), with a value of 16.6 mg GAE/g sample, and F5 (70% tomato powder), with a value of 31.06 mg GAE/g sample. Increasing the concentration of apical tomato powder in seasoning formulations resulted, in general, in an increase in total phenolic content. When comparing the results with those reported by Yaldiz and Camlica (2022) for selected basil (*Ocimum basilicum*) genotypes, a culinary herb widely used as a spice, it can be observed that the quantitative difference is small between the basil genotypes reported (32–40 mg GAE/g dry weight) and the F5 seasoning formulation. Thus, the use of 70% of apical tomato powder can be considered the most adequate from the point of view of phenolic content.

**FIGURE 2:**
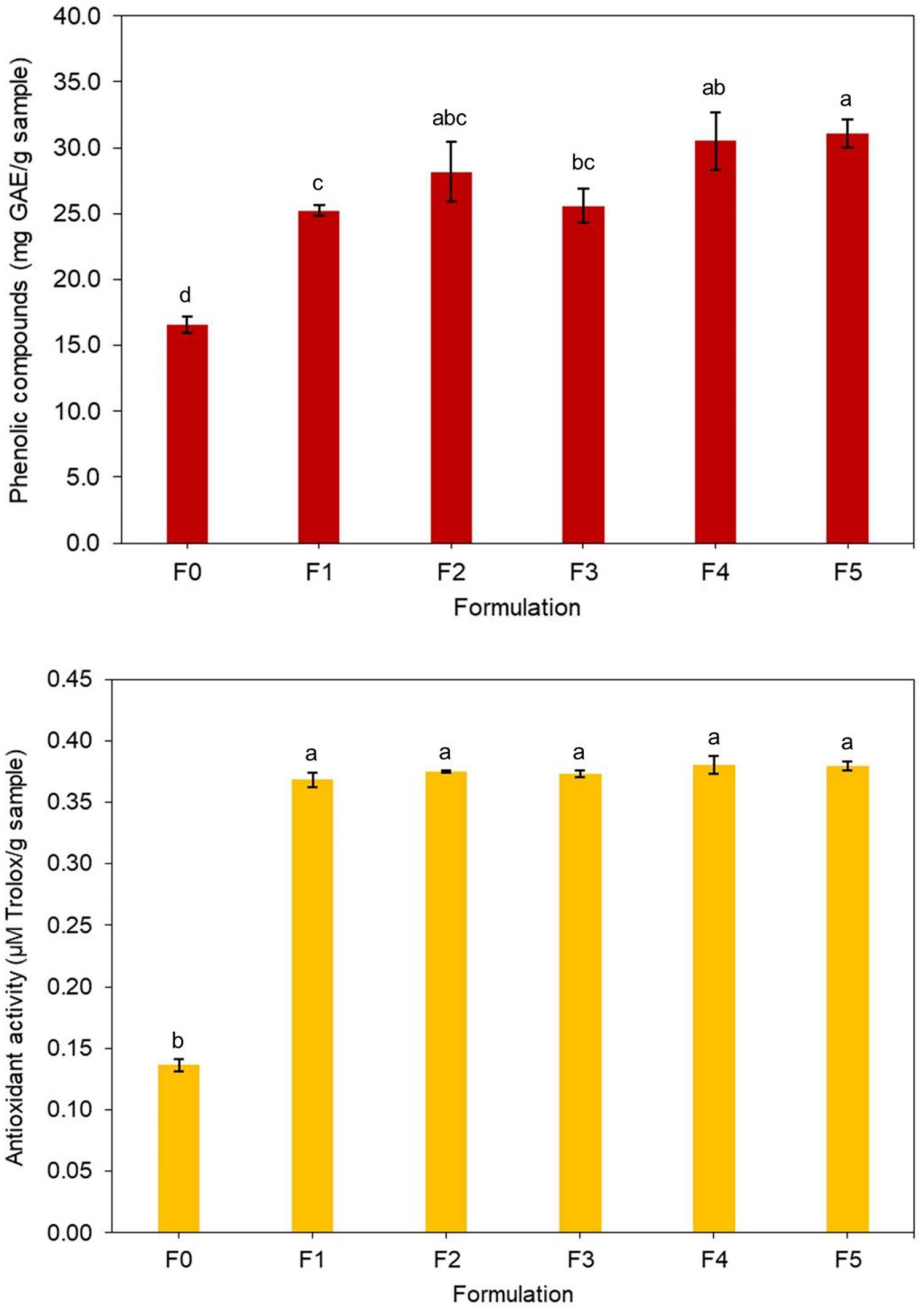
Phenolic compounds and antioxidant activity of apical tomato powder and seasoning formulations (*n* = 3 ± SD). F0 = apical tomato powder; F1 = formulation containing 0% apical tomato; F2 = formulation containing 15% apical tomato; F3 = formulation containing 35% apical tomato; F4 = formulation containing 55% apical tomato; F5 = formulation containing 70% apical tomato. Different letters indicate significant differences (*p* < 0.05) between the samples by Tukey test. GAE, gallic acid equivalent.

Antioxidants are known to help neutralize free radicals in the body and can be found in a variety of foods, especially vegetables and spices (Oliveira et al. 2009). As shown in Figure 2, no significant variation (*p* > 0.05) was observed between the antioxidant activity of the seasoning formulations developed. However, the apical tomato powder showed significantly lower antioxidant activity (*p* < 0.05) than the seasoning samples.

The total phenolic compounds and antioxidant results show that the seasoning produced, besides adding flavor to prepared foods, also promotes health benefits. Such functional profile might be associated with the other compounds used in the production of the seasoning formulations, besides the apical tomato powder. As mentioned in Section 2, the seasoning formulations were produced using paprika, ascorbic acid, garlic, onion, black pepper, red chili pepper, and herbs (rosemary, basil, and oregano), also known for having active properties.

### 3.4 Colorimetry

Colorimetry is a technique that quantifies color by measuring the three primary components of visible light: red, green, and blue. Color is one of the first attributes perceived by the consumer and has a close relationship with product acceptance (Seibel, Kato and Lima 2002).

Color evaluation in the apical tomato, as well as in the seasoning formulations, showed significant differences (*p* < 0.05) between the samples (Table 2). Lightness (*L*) values decreased with the increase in the amount of apical tomato in the seasoning formulations. Values were statistically equal (*p* > 0.05) for tomato powder (F0) and the samples with the highest concentrations (F4 and F5). A similar behavior was observed for the chromaticity coordinate a*. Although the colors of the samples tended to red, the lowest values were observed in the seasonings with lower amounts of apical tomato. For the chromaticity coordinate b*, the values obtained suggest a color tending to yellow, with statistically similar values (*p* > 0.05) predominating for most samples.

**TABLE 2:** Color of apical tomato powder and seasoning formulations (*n* = 3 ± SD).

Sample	*L*	a*	b*	Δ*E*
F0	45.86 ± 0.37^d^	12.12 ± 0.30^ab^	15.15 ± 0.54^ab^	0
F1	84.94 ± 0.09^a^	5.97 ± 0.09^e^	11.11 ± 0.09^b^	39.76 ± 0.06^a^
F2	74.18 ± 3.83^b^	7.71 ± 0.37^d^	15.43 ± 3.89^ab^	26.52 ± 0.01^b^
F3	63.33 ± 3.54^c^	10.57 ± 0.16^c^	15.77 ± 0.88^ab^	15.52 ± 0.08^c^
F4	24.10 ± 0.54^d^	11.79 ± 0.20^cd^	15.37 ± 0.32^ab^	5.26 ± 0.44^d^
F5	49.62 ± 1.35^d^	13.08 ± 0.96^a^	17.17 ± 1.38^a^	3.25 ± 0.10^e^

*Note*: F0 = apical tomato powder; F1 = formulation containing 0% apical tomato; F2 = formulation containing 15% apical tomato; F3 = formulation containing 35% apical tomato; F4 = formulation containing 55% apical tomato; F5 = formulation containing 70% apical tomato. Different letters in the same column indicate significant differences (*p* < 0.05) between the samples by Tukey test.

The variations in the color results of the samples were also visually observed by the modification of their color due to the increase in apical tomato concentration in the seasoning formulations. Figure 3 demonstrates that there was a predominantly reddish color in samples F3, F4, and F5, which resembled that of the apical tomato powder (F0).

**FIGURE 3:**
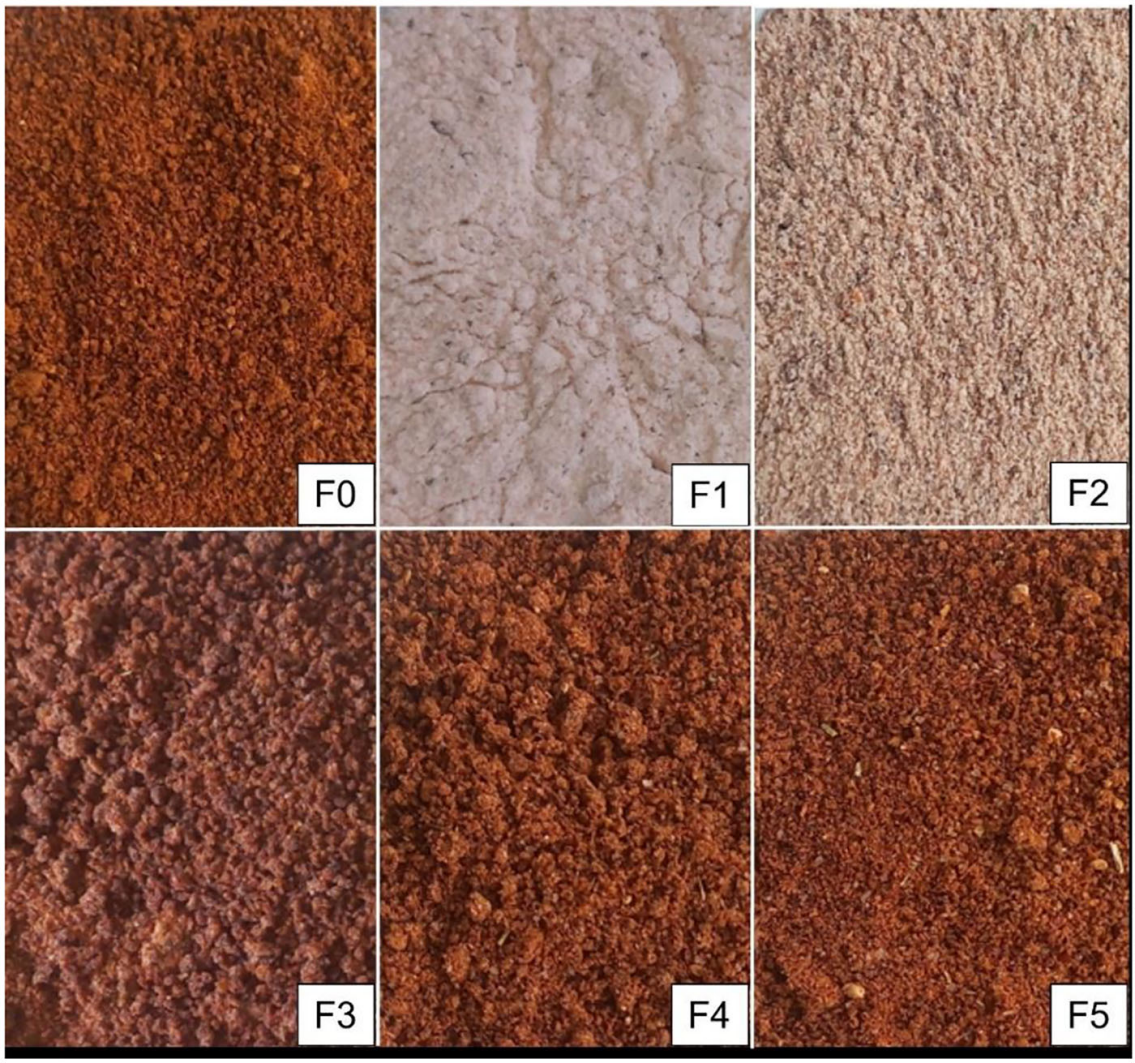
Visual appearance of apical tomato powder and seasoning formulations. F0 = apical tomato powder; F1 = formulation containing 0% apical tomato; F2 = formulation containing 15% apical tomato; F3 = formulation containing 35% apical tomato; F4 = formulation containing 55% apical tomato; F5 = formulation containing 70% apical tomato.

Δ*E* represents the change in all color parameters and is more sensitive to changes compared to *L*, a*, and b*. Significant alterations (*p* < 0.05) were observed for all samples. The highest Δ*E* was observed for F1, whereas the increase in apical tomato powder quantity in the seasoning formulations resulted in a decrease in Δ*E*. According to Lee et al. (2011), Δ*E* > 2.6 represents differences in visual perceptibility. Even though F5 (3.25 ± 0.10) presented the lowest Δ*E*, it could be visually identified as different from the apical tomato powder, corroborating with images presented in Figure 3.

### 3.5 Gas Chromatography–Mass Spectrometry

Gas chromatography is used to separate and quantify various components present in a given sample (Pico et al. 2020). All samples contained compounds that are associated with the aroma of seasonings. Some of them can promote health benefits, such as anti‐inflammatory, antipyretic, antibacterial, and antioxidant. The use of different quantities of apical tomato powder resulted in a specific profile for each formulation, even though emphasis was observed for butanal, 3‐methyl, and eucalyptol.

The sample F0 (100% apical tomato powder) (Table 3) presented mainly organic compounds that are known to contribute specific aromas. The analysis revealed 16 types of compounds, and the most striking was butanal, 3‐methyl, with 79.30% of the spectrum area. In general, this sample has aromas associated with malty, peppery, mushroom‐like, acidic/sourdough‐like, bready, almond‐like, and sweet notes.

**TABLE 3:** Headspace identification by HRGC/MS of the compounds in apical tomato powder (F0).

Peak	RT (min)	AR (%)	Compound
1	2.83	1.26	Furan, 2‐methyl‐
2	3.33	79.3	Butanal, 3‐methyl‐
3	3.67	0.8	Ethanol
4	4.52	1.8	2‐Pentanone
5	4.88	0.41	Undecane
6	7.36	0.27	Hexanal
7	11.07	0.21	Dodecane
8	15.43	4.62	5‐Hepten‐2‐one, 6‐methyl‐
9	15.84	0.22	5‐Heptenal, 2,6‐dimethyl‐
10	18.76	2.57	Acetic acid
11	19.03	6.71	Furfural
12	20.09	0.28	Ethanone, 1‐(2‐furanyl)
13	21.85	0.7	2‐Furancarboxaldehyde, 5‐methyl‐
14	23.90	0.19	2‐Furanmethanol
15	30.52	0.42	Ethanone, 1‐(1H‐pyrrol‐2‐yl)‐
16	32.10	0.25	Glycerol 1,2‐diacetate

Abbreviations: AR, peak area; RT, retention time.

In sample F1 (seasoning with no addition of apical tomato powder), the compounds listed are mainly terpenes and other organic compounds that are common in essential oils and natural flavors. This formulation contains 36 compounds, and the one with the largest area is eucalyptol, with 27.61% of the sample area (Table 4). In this formulation, the aroma is associated with malty, citrusy, and floral to spicy, woody, and sulfurous notes.

**TABLE 4:** Headspace identification by HRGC/MS of the compounds in the F1 formulation (0% apical tomato powder).

Peak	RT (min)	AR (%)	Compound
1	2.961	0.74	Thiirane, methyl‐
2	3.335	2.46	Butanal, 3‐methyl‐
3	3.697	1.22	Ethanol
4	3.958	0.93	Sulfide, allyl methyl
5	5.319	11.38	.alpha.‐Pinene
6	5.523	0.89	Bicyclo[3.1.0]hex‐2‐ene, 2‐methyl‐5‐(1‐methylethyl)
7	6.553	3.16	Camphene
8	7.0	0.32	Disulfide, dimethyl
9	7.649	1.44	Bicyclo[3.1.1]heptane, 6,6‐dimethyl‐2‐methylen
10	8.25	3.19	Bicyclo[3.1.0]hexane, 4‐methylene‐1‐(1‐methylethyl)
11	8.597	3.93	2‐Propen‐1‐ol
12	9.11	0.42	*p*‐Xylene
13	9.312	1.07	Diallyl sulfide
14	9.758	0.94	Bicyclo[3.1.0]hex‐2‐ene, 2‐methyl‐5‐(1‐methylethyl)
15	9.942	1.12	.beta.‐Myrcene
16	10.254	4.01	1,3‐Cyclohexadiene, 1‐methyl‐4‐(1‐methylethyl)
17	10.883	2.49	d‐Limonene
18	11.167	27.61	Eucalyptol
19	12.255	0.21	*Trans*‐.beta.‐ocimene
20	12.424	5.14	.gamma.‐Terpinene
21	13.239	2.38	*o*‐Cymene
22	13.601	2.1	Disulfide, methyl 2‐propenyl
23	16.453	0.84	Dimethyl trisulfide
24	18.801	0.42	Acetic acid
25	19.284	1.53	Diallyl disulfide
26	20.113	5.09	(+)‐2‐Bornanone
27	21.121	0.87	Linalool
28	21.298	0.29	Linalyl acetate
29	21.450	0.47	4‐Isopropyl‐1‐methylcyclohex‐2‐enol
30	22.08	3.25	Caryophyllene
31	22.412	3.45	3‐Cyclohexen‐1‐ol, 4‐methyl‐1‐(1‐methylethyl)
32	23.053	0.22	2‐Cyclohexen‐1‐ol, 1‐methyl‐4‐(1‐methylethyl)‐,
33	24.04	3.08	Estragole
34	24.705	1.87	Isobornyl formate
35	26.676	0.9	Trisulfide, di‐2‐propenyl
36	33.453	0.55	Phenol, 2‐methyl‐5‐(1‐methylethyl)‐

Abbreviations: AR, peak area; RT, retention time.

A variety of aromas that resemble different types of aromas found in nature was presented in formulation F2 (with 15% apical tomato powder). In this sample, 40 compounds were found, and the most prominent in terms of area were butanal, 3‐methyl with area of 11.07%, and eucalyptol with 28.30% (Table 5). These compounds vary widely in their aromatic profiles, which can be minty, sweet, malty, citrusy, among others.

**TABLE 5:** Headspace identification by HRGC/MS of the compounds in F2 (15% tomato powder).

Peak	RT (min)	AR (%)	Compound
1	2.966	0.68	Thiirane, methyl‐
2	3.339	11.07	Butanal, 3‐methyl‐
3	3.7	1.15	Ethanol
4	3.966	0.56	Sulfide, allyl methyl
5	5.319	9.47	.alpha.‐Pinene
6	5.516	0.64	Bicyclo[3.1.0]hex‐2‐ene, 2‐methyl‐5‐(1‐methylethyl)
7	6.555	2.38	Camphene
8	7.612	1.64	Bicyclo[3.1.1]heptane, 6,6‐dimethyl‐2‐methylene
9	8.181	1.93	Bicyclo[3.1.0]hexane, 4‐methylene‐1‐(1‐methylethyl)
10	8.659	3.03	2‐Propen‐1‐ol
11	9.273	0.67	Diallyl sulfide
12	9.706	0.84	Bicyclo[3.1.0]hex‐2‐ene, 2‐methyl‐5‐(1‐methylethyl)
13	9.893	0.96	.beta.‐Myrcene
14	10.202	4.29	1,3‐Cyclohexadiene, 1‐methyl‐4‐(1‐methylethyl)
15	10.839	1.77	d‐Limonene
16	11.149	28.3	Eucalyptol
17	12.232	0.24	*Trans*‐.beta.‐ocimene
18	12.393	5.53	.gamma.‐Terpinene
19	13.217	2.36	*o*‐Cymene
20	13.542	1.2	Cyclohexene, 1‐methyl‐4‐(1‐methylethylidene)‐
21	13.605	0.56	Disulfide, methyl 2‐propenyl
22	15.435	1.08	5‐Hepten‐2‐one, 6‐methyl‐
23	16.452	0.34	Dimethyl trisulfide
24	18.783	1.27	Acetic acid
25	19.054	1.49	Furfural
26	19.283	0.97	Diallyl disulfide
27	20.113	2.5	(+)‐2‐Bornanone
28	21.121	1.27	Linalool
29	21.297	0.35	Linalyl acetate
30	21.452	0.42	4‐Isopropyl‐1‐methylcyclohex‐2‐enol
31	22.083	2.74	Caryophyllene
32	22.19	0.14	Benzene, 2‐methoxy‐4‐methyl‐1‐(1‐methylethyl)
33	22.415	3.23	3‐Cyclohexen‐1‐ol, 4‐methyl‐1‐(1‐methylethyl)‐,
34	23.057	0.23	2‐Cyclohexen‐1‐ol, 1‐methyl‐4‐(1‐methylethyl)
35	24.044	1.53	Estragole
36	24.708	1.75	Isobornyl formate
37	26.678	0.56	Trisulfide, di‐2‐propenyl
38	33.454	0.47	Thymol
39	34.28	0.18	Isospathulenol
40	35.521	0.21	Diethyl phthalate

Abbreviations: AR, peak area; RT, retention time.

In the use of 35% apical tomato powder in F3 (Table 6), the aromatic compounds are mainly terpenes and related compounds, which are found in a variety of natural aromas. This sample contains 39 compounds, and those with the largest areas are butanal, 3‐methyl with 33.17% of area, eucalyptol with 19.67% of area, and .alpha.‐pinene with 7.33% of area. In general, it also has an aroma associated with minty, sweet, malty, citrusy notes, among others.

**TABLE 6:** Headspace identification by HRGC/MS of the compounds in F3 (35% tomato powder).

Peak	RT (min)	AR (%)	Compound
1	2.963	0.84	Thiirane, methyl‐
2	3.338	33.17	Butanal, 3‐methyl‐
3	3.685	1.71	Ethanol
4	3.955	0.51	Sulfide, allyl methyl
5	5.324	7.33	.alpha.‐Pinene
6	5.525	0.45	Bicyclo[3.1.0]hex‐2‐ene, 2‐methyl‐5‐(1‐methylethyl)
7	6.561	1.7	Camphene
8	7.636	0.64	Bicyclo[3.1.1]heptane, 6,6‐dimethyl‐2‐methyle
9	8.221	0.86	Bicyclo[3.1.0]hexane, 4‐methylene‐1‐(1‐methylethyl
10	8.637	2.61	2‐Propen‐1‐ol
11	9.297	0.55	Diallyl sulfide
12	9.739	0.6	Bicyclo[3.1.0]hex‐2‐ene, 2‐methyl‐5‐(1‐methylethyl)
13	9.921	0.94	.beta.‐Myrcene
14	10.232	3.12	1,3‐Cyclohexadiene, 1‐methyl‐4‐(1‐methylethyl)
15	10.864	1.26	d‐Limonene
16	11.160	19.67	Eucalyptol
17	12.411	3.59	.gamma.‐Terpinene
18	13.230	1.55	Benzene, 1‐methyl‐3‐(1‐methylethyl)‐
19	13.553	1.28	Cyclohexene, 1‐methyl‐4‐(1‐methylethylidene)‐
20	15.437	2.43	5‐Hepten‐2‐one, 6‐methyl‐
21	16.306	0.08	Cyclohexasiloxane, dodecamethyl‐
22	16.459	0.28	Dimethyl trisulfide
23	18.6	0.07	1‐Octen‐3‐ol
24	18.781	1.29	Acetic acid
25	19.046	3.33	Furfural
26	19.283	0.68	Diallyl disulfide
27	20.112	1.84	(+)‐2‐Bornanone
28	20.746	0.07	Cycloheptasiloxane, tetradecamethyl‐
29	21.122	0.57	Linalool
30	21.285	0.12	Linalyl acetate
31	21.863	0.33	2‐Furancarboxaldehyde, 5‐methyl‐
32	22.083	1.7	Caryophyllene
33	22.415	1.8	3‐Cyclohexen‐1‐ol, 4‐methyl‐1‐(1‐methylethyl)
34	24.146	0.91	Estragole
35	24.707	1.09	.alpha.‐Terpineol
36	26.677	0.33	Trisulfide, di‐2‐propenyl
37	30.533	0.18	Ethanone, 1‐(1H‐pyrrol‐2‐yl)‐
38	33.455	0.3	Phenol, 2‐methyl‐5‐(1‐methylethyl)‐
39	35.524	0.24	Diethyl phthalate

Abbreviations: AR, peak area; RT, retention time.

In sample F4 (55% apical tomato powder), 43 compounds were found, and the most striking were butanal, 3‐methyl with 49.79% of area, eucalyptol with 9.14% of area, and furfural with 5.90% of area (Table 7). These compounds are found in many plants and natural products, contributing to a variety of aromas, ranging from citrusy and minty to sweet, malty, bready, and almond‐like.

**TABLE 7:** Headspace identification by HRGC/MS of the compounds in F4 (55% apical tomato powder).

Peak	RT (min)	AR (%)	Compound
1	2.83	0.48	Furan, 2‐methyl‐
2	2.974	1.29	Thiirane, methyl‐
3	3.339	49.79	Butanal, 3‐methyl
4	3.683	0.75	Ethanol
5	4.525	0.95	2‐Pentanone
6	5.32	2.6	.alpha.‐Pinene
7	6.558	0.52	Camphene
8	7.001	0.18	Disulfide, dimethyl
9	7.611	0.14	Bicyclo[3.1.1]heptane, 6,6‐dimethyl‐2‐methylene
10	8.146	0.55	Bicyclo[3.1.0]hexane, 4‐methylene‐1‐(1‐methylethyl)
11	8.707	1.9	2‐Propen‐1‐ol
12	9.06	0.24	Benzene, (2‐methoxyethyl)‐
13	9.251	0.32	Diallyl sulfide
14	9.673	0.43	Bicyclo[3.1.0]hex‐2‐ene, 2‐methyl‐5‐(1‐methylethyl)
15	9.862	0.47	.beta.‐Myrcene
16	10.171	2.17	1,3‐Cyclohexadiene, 1‐methyl‐4‐(1‐methylethyl)
17	10.813	0.66	d‐Limonene
18	11.138	9.14	Eucalyptol
19	12.376	2.7	.gamma.‐Terpinene
20	13.105	0.18	Propane, 1‐(methylthio)‐
21	13.207	0.92	*o*‐Cymene
22	13.526	0.5	(+)‐4‐Carene
23	13.588	0.49	Disulfide, methyl 2‐propenyl
24	15.433	3.4	5‐Hepten‐2‐one, 6‐methyl‐
25	16.307	0.15	Cyclohexasiloxane, dodecamethyl‐
26	18.764	3.93	Acetic acid
27	19.041	5.9	Furfural
28	19.276	0.61	Diallyl disulfide
29	20.109	0.7	Cyclopropane, 2‐(1,1‐dimethyl‐2‐pentenyl)‐1,1‐
30	20.750	0.45	Cycloheptasiloxane, tetradecamethyl‐
31	21.124	0.42	Linalool
32	21.859	0.69	2‐Furancarboxaldehyde, 5‐methyl‐
33	22.079	1.59	Caryophyllene
34	22.416	1.22	3‐Cyclohexen‐1‐ol, 4‐methyl‐1‐(1‐methylethyl)‐,
35	23.908	0.2	2‐Furanmethanol
36	24.045	0.99	Estragole
37	24.738	0.79	Isoborneol
38	26.679	0.35	Trisulfide, di‐2‐propenyl
39	30.526	0.45	Ethanone, 1‐(1H‐pyrrol‐2‐yl)‐
40	33.455	0.23	Thymol
41	34.464	0.37	4H‐Pyran‐4‐one, 2,3‐dihydro‐3,5‐dihydroxy‐6‐
42	35.525	0.23	Diethyl Phthalate
43	36.773	0.27	5‐Hydroxymethylfurfural

Abbreviations: AR, peak area; RT, retention time.

The higher quantity of apical tomato powder (70% in F5) showed compounds associated with a wide variety of aromas. Many of these are found in plants, herbs, fruits, and other organic materials. In this analysis, 41 compounds were found, and the largest area is occupied by butanal, 3‐methyl with 51.82% of area, followed by eucalyptol with 10.95% of area (Table 8), similar to the other formulations containing higher quantities of apical tomato powder. These compounds are also found in a diverse range of products such as essential oils, herbs, spices, fruits, and food products.

**TABLE 8:** Headspace identification by HRGC/MS of the compounds in F5 (70% apical tomato powder).

Peak	RT (min)	AR (%)	Compound
1	2.839	0.52	Furan, 2‐methyl‐
2	2.956	0.99	Thiirane, methyl‐
3	3.339	51.82	Butanal, 3‐methyl‐
4	3.703	0.99	Ethanol
5	3.956	0.34	Sulfide, allyl methyl
6	4.524	1.1	2‐Pentanone
7	5.324	5.28	.alpha.‐Pinene
8	5.523	0.33	Bicyclo[3.1.0]hex‐2‐ene, 2‐methyl‐5‐(1‐methylethyl)
9	6.561	1.14	Camphene
10	6.999	0.24	Disulfide, dimethyl
11	7.365	1.24	Hexanal
12	7.629	0.53	Bicyclo[3.1.1]heptane, 6,6‐dimethyl‐2‐methylene
13	8.209	0.55	Bicyclo[3.1.0]hexane, 4‐methylene‐1‐(1‐methylethyl)
14	8.646	1.55	2‐Propen‐1‐ol
15	9.292	0.28	Diallyl sulfide
16	9.728	0.34	.alpha.‐Phellandrene
17	9.913	0.69	.beta.‐Myrcene
18	10.223	1.94	1,3‐Cyclohexadiene, 1‐methyl‐4‐(1‐methylethyl)
19	10.859	0.78	d‐Limonene
20	11.159	10.95	Eucalyptol
21	12.407	2.27	.gamma.‐Terpinene
22	13.115	0.12	Propane, 1‐(methylthio)‐
23	13.229	0.92	*o*‐Cymene
24	13.556	0.96	Cyclohexene, 1‐methyl‐4‐(1‐methylethylidene)
25	15.439	3.06	5‐Hepten‐2‐one, 6‐methyl‐
26	15.849	0.17	5‐Heptenal, 2,6‐dimethyl‐
27	18.774	1.55	Acetic acid
28	19.043	4.13	Furfural
29	19.286	0.37	Diallyl disulfide
30	20.117	1.48	Camphor
31	21.127	0.33	Linalool
32	21.862	0.47	2‐Furancarboxaldehyde, 5‐methyl‐
33	22.088	0.88	Caryophyllene
34	22.421	0.77	3‐Cyclohexen‐1‐ol, 4‐methyl‐1‐(1‐methylethyl)‐,
35	23.909	0.11	2‐Furanmethanol
36	24.050	0.68	Estragole
37	24.712	0.4	.alpha.‐Terpineol
38	26.682	0.17	Trisulfide, di‐2‐propenyl
39	30.528	0.26	Ethanone, 1‐(1H‐pyrrol‐2‐yl)‐
40	33.455	0.12	Phenol, 2‐methyl‐5‐(1‐methylethyl)‐
41	35.525	0.15	Diethyl phthalate

Abbreviations: AR, peak area; RT, retention time.

### 3.6 Structure and Morphology

The structures and morphologies of the apical tomato powder and the seasoning formulations were visualized through SEM, which reveals the composition and topography of the analyzed element. The micrograph of the apical tomato powder shows an irregular surface with different sizes and agglomerated shapes (Figure 4). On the other hand, the micrographic visualization of seasoning F1 (0% apical tomato powder) shows a sample with irregular shapes, with some larger and more defined particles, whereas others are smaller and fragmented, and some particles are more agglomerated and others more isolated. In sample F2 (15% apical tomato powder), the shapes are irregular, but some are similar, with larger and more defined particles, some with a porous aspect, and others have a smooth appearance and are dispersed. A structure with a hollow conical appearance was also visualized. Sample F3 (35% apical tomato powder) contains particles with different shapes and sizes, fragmented and agglomerated, showing an irregular structure and porous aspect. In sample F4 (55% apical tomato powder), the particles are non‐uniform, with different shapes and sizes, some with a smooth and shiny appearance and other fragments apparently compacted into some resembling “clods,” fragments with rough and rugged edges. In the vast majority, the particles are isolated. The micrographs of the F5 sample (70% apical tomato powder) show a structure with irregular shapes, with different shapes and sizes. Some particles appear to have overlapped layers, and a porous and wrinkled appearance is present in others, in addition to uneven agglomeration, as the particles are closer to each other, forming small clusters, whereas others are more isolated.

**FIGURE 4:**
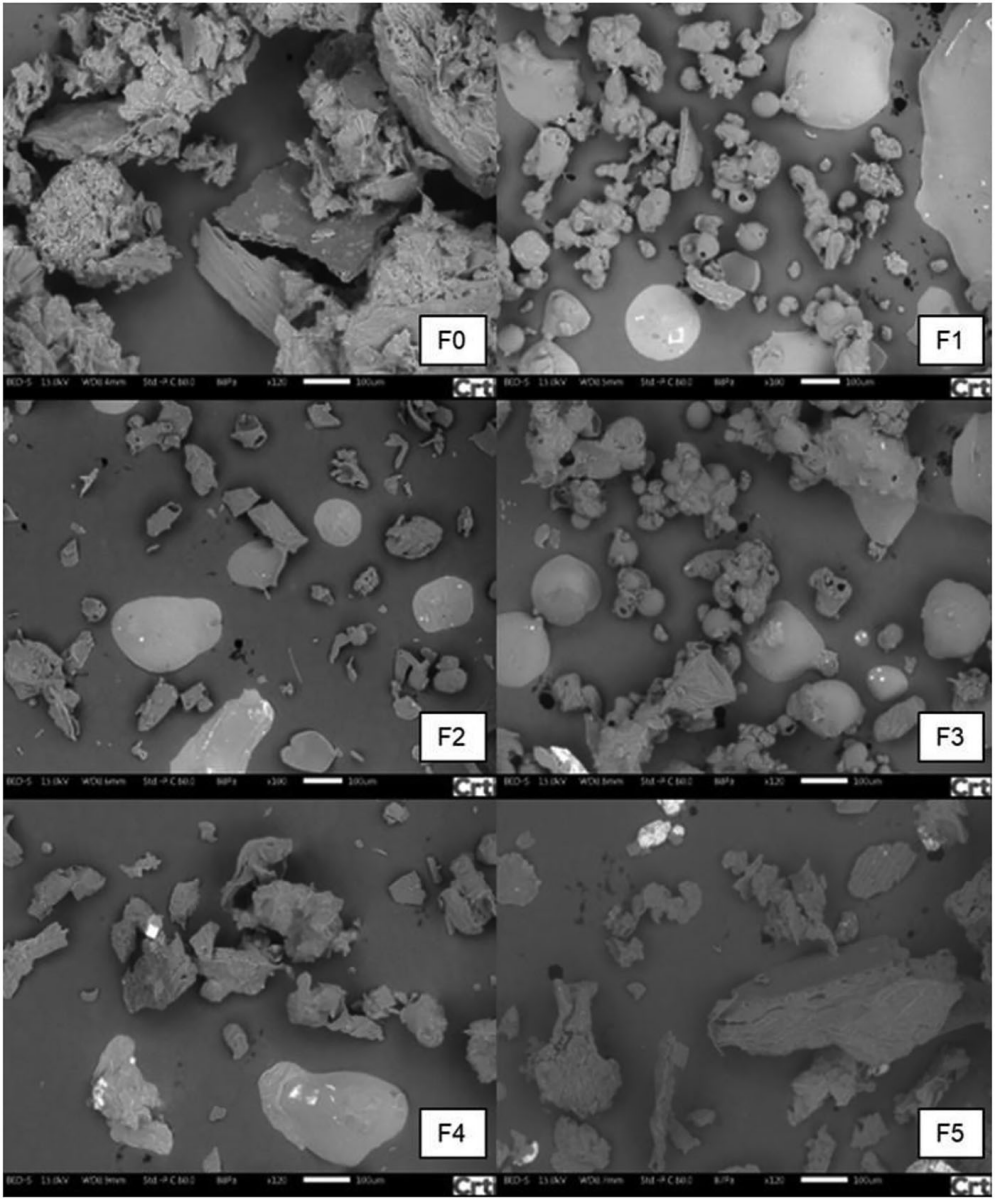
Micrographs of the structures and morphologies of apical tomato powder and seasoning formulations. F0 = apical tomato powder (×120); F1 = formulation containing 0% apical tomato (×100); F2 = formulation containing 15% apical tomato (×120); F3 = formulation containing 35% apical tomato (×120); F4 = formulation containing 55% apical tomato (×120); F5 = formulation containing 70% apical tomato (×120).

In general, all samples showed uneven particles, with different shapes, sizes, and agglomerations. This pattern results from the characteristics of the components used to make the seasonings, in addition to the apical tomato powder. The agglomeration of the particles may be associated with the previously discussed hygroscopic degree of the samples, as reported in the literature (Bhandari et al. 2013).

### 3.7 Microbiological Analysis

ANVISA's NI No. 161 of July 01, 2022 establishes the microbiological standards of food. The apical tomato‐based seasoning produced here is included in Category 18, Item b—spices, seasonings, and sauces—dehydrated, pasted, or pressed seasonings (bean seasoning mix, bouillon cubes, sauté mixes), which requires analyses to detect *E. coli* and *Salmonella* spp.

Microbiological analysis of the F0 formula was not performed because this sample consists only of the apical tomato powder, so the definition of seasoning does not apply, as seasonings are products obtained from the mixture of spices and other ingredient(s), fermented or not, used to add flavor or aroma to foods and beverages, according to RDC No. 276, of September 22, 2005.

Table 9 presents the results of the microbiological analysis performed on the seasoning formulations developed. The results indicate that the presence of *E. coli* and *Salmonella* spp. was not detected in the samples within the minimum limits quantified by the analysis. The results obtained are satisfactory, as they indicate that the products are safe for consumption and that they are within the compliance standards required by Brazilian legislation.

**TABLE 9:** Microbiological analysis of seasoning formulations containing apical tomatoes.

Sample	Microorganism	Result	Reference values^a^
F1	*Escherichia coli*	<1.0 × 10^1^ CFU/g	*n* = 1; *m* = 1.0 × 10^1^; *M* = 1.0 × 10^2^
	*Salmonella* spp.	Absence	Absence in 25 g
F2	*Escherichia coli*	<1.0 × 10^1^ CFU/g	*n* = 1; *m* = 1.0 × 10^1^; *M* = 1.0 × 10^2^
	*Salmonella* spp.	Absence	Absence in 25 g
F3	*Escherichia coli*	<1.0 × 10^1^ CFU/g	*n* = 1; *m* = 1.0 × 10^1^; *M* = 1.0 × 10^2^
	*Salmonella* spp.	Absence	Absence in 25 g
F4	*Escherichia coli*	<1.0 × 10^1^ CFU/g	*n* = 1; *m* = 1.0 × 10^1^; *M* = 1.0 × 10^2^
	*Salmonella* spp.	Absence	Absence in 25 g
F5	*Escherichia coli*	<1.0 × 10^1^ CFU/g	*n* = 1; *m* = 1.0 × 10^1^; *M* = 1.0 × 10^2^
	*Salmonella* spp.	Absence	Absence in 25 g

*Note*: F1 = formulation containing 0% apical tomato; F2 = formulation containing 15% apical tomato; F3 = formulation containing 35% apical tomato; F4 = formulation containing 55% apical tomato; F5 = formulation containing 70% apical tomato.

^a^Reference values according to ANVISA's Normative Instruction No. 161 of July 01, 2022.

Seasoning is a dry product (the water activity is low—0.20–0.60, and microorganisms need at least 0.80 for growth) (Gurtler and Keller 2019), explaining that the formulations produced are expected to be microbiologically stable over time. It is also important to point out that the formulations have salt exerting preservative/antimicrobial effects, and the herbs and spices used are known to contain compounds with antimicrobial activity (Tajkarimi et al. 2010), which might contribute to seasoning preservation over time.

### 3.8 Sensory Analysis

In the sensory analysis, only the seasoning formulations developed were evaluated, as the apical tomato powder is one of the ingredients used. The results of the sensory analysis presented in Table 10 showed that the seasoning had a significant improvement as it progressed from F1 (0% apical tomato powder) to F5 (70% apical tomato powder). This progression suggests that increasing the percentages of apical tomato powder in the seasoning had a positive effect on the consumers’ acceptance. This effect was probably associated with the color of the formulations, which became more reddish (Figure 3) as the concentration of apical tomato powder increased. Formulations F4 and F5 had the best averages in all the evaluated attributes, with values that were, in general, significantly superior (*p* < 0.05) to the others, indicating high acceptance of the product and market potential. This was confirmed by the evaluation of purchase intention; seasoning formulations containing higher quantities of apical tomato powder (F4 and F5) presented average values around 4, which were significantly superior (*p* < 0.05) to the other formulations. Such results point to these formulations as a product with a positive sign for launch in the market.

**TABLE 10:** Results of sensory analysis of seasoning formulations containing apical tomato.

Hedonic scale						
Sample	Appearance	Odor	Texture	Taste	Overall rating	Purchase intention
F1	5.64 ± 1.78^d^	6.02 ± 1.45^c^	6.39 ± 1.67^b^	5.79 ± 1.76^c^	5.95 ± 1.55^c^	2.76 ± 1.20^c^
F2	6.50 ± 1.62^c^	6.35 ± 1.57^bc^	6.80 ± 1.55^ab^	6.42 ± 1.72^bc^	6.57 ± 1.52^b^	3.34 ± 1.21^b^
F3	7.15 ± 1.44^b^	6.52 ± 1.40^abc^	6.81 ± 1.60^ab^	6.63 ± 1.73^ab^	6.89 ± 1.51^ab^	3.48 ± 1.11^b^
F4	7.52 ± 1.20^ab^	6.89 ± 1.24^ab^	7.14 ± 1.36^a^	7.15 ± 1.35^a^	7.23 ± 1.34^a^	3.98 ± 1.02^a^
F5	7.81 ± 1.24^a^	7.03 ± 1.39^a^	7.20 ± 1.26^a^	7.02 ± 1.52^ab^	7.37 ± 1.22^a^	4.00 ± 1.10^a^

*Note*: F1 = formulation containing 0% apical tomato; F2 = formulation containing 15% apical tomato; F3 = formulation containing 35% apical tomato; F4 = formulation containing 55% apical tomato; F5 = formulation containing 70% apical tomato. Different letters in the same column indicate significant differences (p < 0.05) between the samples by Tukey test.

## 4 Conclusion

Table tomato production has great potential for the manufacture of products with added value, as in the case of the seasoning developed in the present study. Apical tomato‐based seasonings represent an opportunity to value tomatoes that would be commonly discarded, combining the reduction of food waste with the development of natural, functional, sustainable, and sensorially attractive products. All formulations showed promising results, as demonstrated through the analyses carried out. Formulation F5 stood out in the amount of phenolic compounds, with 31.06 mg GAE/g sample and antioxidant activity of 0.38 µM/g sample. The color of this sample tended to be dark, red, and yellow, which probably led to its greater acceptance and purchase intention. On the other hand, it was also the sample that had the highest hygroscopicity, indicating the need for adequate packaging and storage conditions. As for aromatic compounds, this formulation had the second largest quantity of compounds, with 40 components. In the microbiological analysis, all samples showed satisfactory results, as all were within the standards of the legislation. The micrographs obtained in SEM showed that each sample had a specific structure, but in general, the structures were agglomerated, with different sizes and shapes. Therefore, F5 is suggested as the most promising for future studies; it is important to complement its characterization with water activity (Aw), moisture content, acidity, and pH measurements. Also, it is fundamental to evaluate its stability throughout storage, estimating its shelf life and potential deterioration mechanisms—including microbiological, physicochemical, sensory, and physical changes, as well as appropriate critical limits for defining the end of the product's shelf life. Besides that, to determine the most appropriate packaging material to maintain the quality attributes of the seasoning until its consumption. It is suggested to study shelf life under different temperature conditions and packaging materials for approximately 4–6 months.

## Author Contributions

**Márcia Aparecida de Souza**: conceptualization, methodology, writing – original draft, formal analysis, data curation. **Maria Gláucia Dourado Furquim**: conceptualization, investigation, supervision, writing – review and editing. **Viviane Patrícia Romani**: writing – review and editing, data curation, visualization. **Julio Colivet Briceno**: conceptualization, investigation, methodology, visualization. **Bruna Melo Miranda**: formal analysis, methodology, investigation. **Tatianne Ferreira de Oliveira**: supervision, visualization, project administration, methodology. **Abadia dos Reis Nascimento**: conceptualization, resources, supervision, project administration, funding acquisition.

## Conflicts of Interest

The authors declare no conflicts of interest.
